# Genome-Wide Identification and Characterization of *OSC* Gene Family in *Gynostemma pentaphyllum* (Cucurbitaceae)

**DOI:** 10.3390/life14121599

**Published:** 2024-12-04

**Authors:** Xiao Zhang, Huan Yang, Xuan Wang, Xiaoting Wang, Chen Chen

**Affiliations:** 1 Xi’an Botanical Garden of Shaanxi Province, Institute of Botany of Shaanxi Province, No. 17 Cuihua South Road, Xi’an 710061, China; zhxiaao@163.com (X.Z.); wx36134@163.com (X.W.); 15996245235@163.com (X.W.); 2 Shaanxi Engineering Research Centre for Conservation and Utilization of Botanical Resources, No. 17 Cuihua South Road, Xi’an 710061, China; 3 College of Life Sciences, Northwest University, Xi’an 710069, China; yanghuanmy@stumail.nwu.edu.cn; 4 School of Life Science, Shaanxi Normal University, Xi’an 710119, China

**Keywords:** *Gynostemma pentaphyllum*, triterpene saponins, 2,3-oxidized squalene cyclase, *OSC* gene family, gene expression

## Abstract

*Gynostemma pentaphyllum* is a traditional Chinese medicinal plant of considerable application value and commercial potential, primarily due to its production of various bioactive compounds, particularly dammarane-type triterpenoid saponins that are structurally analogous to ginsenosides. Oxidosqualene cyclase (OSC), a pivotal enzyme in the biosynthesis of triterpenoid metabolites in plants, catalyzes the conversion of oxidosqualene into triterpenoid precursors, which are essential components of the secondary metabolites found in *G. pentaphyllum*. To elucidate the role of *OSC* gene family members in the synthesis of gypenosides within *G. pentaphyllum*, this study undertook a comprehensive genome-wide identification and characterization of *OSC* genes within *G. pentaphyllum* and compared their expression levels across populations distributed over different geographical regions by both transcriptome sequencing and qRT-PCR experimental validation. The results identified a total of 11 members of the *OSC* gene family within the genome of *G. pentaphyllum*. These genes encode proteins ranging from 356 to 767 amino acids, exhibiting minor variations in their physicochemical properties, and are localized in peroxisomes, cytoplasm, plasma membranes, and lysosomes. All *GpOSC*s contain highly conserved DCTAE and QW sequences that are characteristic of the *OSC* gene family. A phylogenetic analysis categorized the *GpOSC*s into four distinct subfamilies. A cis-element analysis of the *GpOSC* promoters revealed a substantial number of abiotic stress-related elements, indicating that these genes may respond to drought conditions, low temperatures, and anaerobic environments, thus potentially contributing to the stress resistance observed in *G. pentaphyllum*. Expression analyses across different *G. pentaphyllum* populations demonstrated significant variability in *OSC* gene expression among geographically diverse samples of *G. pentaphyllum*, likely attributable to genetic variation or external factors such as environmental conditions and soil composition. These differences may lead to the synthesis of various types of gypenosides within geographically distinct *G. pentaphyllum* populations. The findings from this study enhance our understanding of both the evolutionary history of the *OSC* gene family in *G. pentaphyllum* and the biosynthetic mechanisms underlying triterpenoid compounds. This knowledge is essential for investigating molecular mechanisms involved in forming dammarane-type triterpenoid saponins as well as comprehending geographical variations within *G. pentaphyllum* populations. Furthermore, this research lays a foundation for employing plant genetic engineering techniques aimed at increasing gypenoside content.

## 1. Introduction

*Gynostemma pentaphyllum* (Thunb.) Makino, a member of the Cucurbitaceae family, is a perennial creeping herbaceous plant. This species is predominantly distributed in tropical Asia, particularly in the southern regions of the Qinling Mountains and the moist forests of the Yangtze River Basin in China [1]. As a traditional Chinese medicinal plant, *G. pentaphyllum* is commonly referred to as “Southern ginseng” and possesses significant medicinal value due to its content of ginsenosides and other structurally similar dammarane-type saponins [2]. Furthermore, *G. pentaphyllum* contains various bioactive compounds including saponins, flavonoids, amino acids, and vitamins [3]. Among these constituents, gypenosides are recognized as the primary active ingredients, which exhibit antioxidant properties along with anti-cancer effects, lipid-lowering capabilities, and immune-enhancing activities [4,5,6]. Currently, *G. pentaphyllum* has been extensively developed into health care products such as oral liquids, health capsules, and teas, which present vast commercial potential [7]. In recent years, there has been growing interest in developing drugs and health foods containing gypenosides; this trend presents both unprecedented opportunities and challenges for harnessing *G. pentaphyllum* resources.

In 1981, dammarane-type saponin components (panaxadiol and 2α-hydroxypanaxadiol) were first isolated from *G. pentaphyllum* [8]. These compounds bear structural similarities to ginsenosides and have garnered considerable attention from researchers. Since then, numerous studies have yielded significant findings regarding the principal chemical constituents of *G. pentaphyllum*, particularly concerning its saponin compounds. To date, over 200 distinct saponins have been isolated from this plant; notably, about 25% share structures akin to those found in ginsenosides [7]. Importantly, gypenosides III, IV, VIII, XIII, I, and A-AH possess identical structures to their counterparts among ginsenosides Rb1, Rb3, Rd, F2, K, and Rg3, respectively, and they have thus been identified as equivalent substances under different nomenclatures [9]. Due to the high demand for ginsenosides in the pharmaceutical industry, gypenosides can be converted into ginsenosides through bacterial enzyme engineering technology. This has led to increased interest in *G. pentaphyllum* within medicinal plant research [10]. Furthermore, the genus *Gynostemma* is the only taxon besides *Panax* that contains ginsenosides. It offers numerous advantages such as wide distribution, rapid growth, and a higher saponin content, rendering it valuable for both practical applications and academic research [11,12,13]. However, the type and content of gypenosides are often closely associated with the geographical distribution of *G. pentaphyllum* [14]. Consequently, investigating the content of gypenosides and their biosynthetic genes across different regions inhabited by *G. pentaphyllum* can not only elucidate the molecular mechanisms underlying gypenoside formation, but also provide comprehensive theoretical support for developing high-quality traditional Chinese medicine.

Currently, significant progress has been made in studies concerning the biosynthesis pathway of ginsenosides. However, research on the biosynthesis of gypenosides remains limited. Previous investigations have demonstrated that saponins derived from both *G. pentaphyllum* and *Panax ginseng* exhibit structural similarities and fall under tetracyclic triterpenoid saponins [3,12]. The biosynthetic pathway comprises three primary stages: (1) Initial stage—isopentenyl pyrophosphate (IPP) and dimethylallyl pyrophosphate (DMAPP) are synthesized. (2) Formation stage of triterpenoid skeleton—IPP and DMAPP molecules undergo successive catalysis by geranyl pyrophosphate synthase (GPPS), farnesyl pyrophosphate synthase (FPS), squalene synthase (SS), and squalene epoxidase (SE) to yield 2,3-oxidosqualene. Subsequently, 2,3-oxidosqualene can generate various types of triterpenoid skeletons under the influence of distinct oxidosqualene cyclases (OSCs). (3) Modification stage of triterpenoid skeleton—under the influence of cytochrome P450, glycosyltransferases, acyltransferases, and other modifying enzymes, various triterpenoid saponin end products are generated through oxidation, glycosylation, acylation, and other chemical modifications of the triterpenoid skeletons [11]. In this process, the cyclization of 2,3-oxidosqualene by an OSC enzyme represents a critical initial step in the biosynthesis of plant triterpenoid saponins. This step is pivotal in determining the diversity of triterpenoid products and has the potential to yield over 100 distinct triterpenoid saponin skeletons [15,16].

The OSC (oxidosqualene cyclase) is a key enzyme within the biosynthetic pathway for sterols and triterpenes in plants. Based on intermediate structures formed during substrate binding and folding, namely chair–chair–chair (CCC) and chair–boat–chair (CBC) conformations, sterol and triterpene compounds are synthesized. As a super gene family in plants, the *OSC* family members exhibit highly conserved sequences such as DCTAE and QW (QXXXXXW) [17]. The DCTAE conserved sequence is associated with substrate binding, while the QW characteristic sequence comprises negatively charged aromatic amino acids that play a stabilizing role during the cyclization reaction of 2,3-oxidized squalene [18]. With ongoing investigations into secondary metabolic pathways in plants, an increasing number of *OSC* genes have been isolated, cloned, and functionally validated. To date, more than 200 *OSC* genes have been identified across various plant species. Their catalytic functions have primarily been elucidated through heterologous expression studies conducted in yeast models including *Arabidopsis thaliana* [19], *P. ginseng* [20,21], and *Glycyrrhiza uralensis* [22]. The *OSC* genes found in plants facilitate several reactions involving protonation, cyclization, rearrangement, and deprotonation processes on 2,3-oxidized squalene to produce diverse cyclic compounds such as cycloartenol, lanosterol, dammarandienol, lupeol, α-amyrin, β-amyrin, and other triterpene skeletons [23]. However, the structures and reaction mechanisms of various types of enzymes within the *OSC* gene family remain incompletely understood. Consequently, the identification and analysis of *OSC* family genes in medicinal plants, along with investigations into diverse OSC catalytic mechanisms, represent current trends and challenges for this gene family.

In contrast to model plants, such as *Arabidopsis* and rice, medicinal plants encompass a vast array of species, many of which possess unclear genetic backgrounds due to a lack of genomic and transcriptomic data as well as limited research on their functional genes. This deficiency significantly impedes advancements in the molecular biology of medicinal plants [24,25]. Presently, more and more modern biotechnological methods are widely used in the molecular research of medicinal plants. Notably, next-generation high-throughput RNA sequencing technology is extensively utilized for transcriptome analyses and functional gene studies [26,27,28]. By examining variations in gene expression levels across different tissues, physiological states, and population distributions, it becomes possible to identify genes and regulatory factors associated with secondary metabolite biosynthesis pathways. This process plays a crucial role in inferring key enzyme genes involved in secondary metabolite biosynthesis within important medicinal plants while elucidating their metabolic pathways and regulatory mechanisms [29].

Benefiting from the previously obtained chromosomal-level whole-genome sequence of *G. pentaphyllum* [30], this study systematically identified and analyzed *OSC* genes across the entire genome. Furthermore, it compared their expression levels in traditional medicinal plant *G. pentaphyllum* from various regions using transcriptome sequencing technology and qRT-PCR experimental validation. The results will contribute to a deeper understanding of the systematic evolution of *OSC* genes and the biosynthesis mechanisms of triterpenoids in *G. pentaphyllum*, which holds significant importance for elucidating the molecular mechanisms underlying dammarane-type triterpenoid saponin formation in *G. pentaphyllum*.

## 2. Materials and Methods

### 2.1. Genome-Wide Identification and Chromosomal Distribution of OSC Gene Family in G. pentaphyllum

To identify members of the *OSC* gene family across the genome of *G. pentaphyllum*, we initially downloaded all known *OSC* genes of *A. thaliana* available in the TAIR library (http://www.Arabidopsis.org/, accessed on 29 December 2023). Additionally, sequences of previously identified *OSC*s from *P. ginseng*, *P. notoginseng*, *P. quiquefolium*, *Luffa cylindrica*, *Cucurbita pepo*, and *Momordica charantia* were retrieved from NCBI (Table S1). Subsequently, BLAST searches were conducted against the database for *G. pentaphyllum* (https://www.ncbi.nlm.nih.gov/datasets/genome/GCA_028015215.1/ [30], accessed on 29 December 2023), utilizing an E-value threshold ≤ 1 × 10^−5^ with data assembled and annotated through the BLASTP online tool [31]. Next, HMMER 3.0 [32] was employed with default parameters to search for *OSC* coding proteins based on hidden Markov models corresponding to both the C-terminal domain (PF13243) and N-terminal domain (PF13249) of squalene-hopene cyclase [17]. Candidate genes containing both domains were validated using Pfam (http://pfam.xfam.org/, accessed on 31 December 2023) as well as the Conserved Domain Database (CDD) at NCBI (https://www.ncbi.nlm.nih.gov/cdd/, E-value of 1 × 10^−5^, other parameters set to defaults, accessed on 31 December 2023 [33]), thereby identifying them as *GpOSC*s. The chromosomal distribution of target *GpOSC* genes within each chromosome of *G. pentaphyllum* was determined by utilizing whole-genome annotation files along with gene density information via the Gene Location Visualizer tool in the GTF/GFF function module of TBtools version 2.096 [34].

### 2.2. Physicochemical Property and Subcellular Localization Analysis of GpOSC Family

The features of GpOSC proteins, including amino acid count, molecular weight (MW), theoretical isoelectric point (pI), and grand average hydropathicity (GRAVY), were calculated using the ExPaSy Proteomics Server (http://www.expasy.org/sprot/sp-docu.html, accessed on 6 January 2024). To predict the subcellular localization of *GpOSC*s, protein sequences of *GpOSC*s were analyzed using the WoLFPSORT program with default parameters (https://wolfpsort.hgc.jp/, accessed on 6 January 2024).

### 2.3. Phylogenetic Analysis

The nucleotide sequences of eleven *GpOSC* genes alongside 84 *OSC* genes from various plant species were downloaded from NCBI (Table S1) and aligned using the MUSCLE alignment package in MEGA version 7.0. The phylogenetic relationships among *OSC* gene families were constructed, employing the neighbor-joining (NJ) method with 1000 bootstraps. The online iTOL tool (https://itol.embl.de/, accessed on 10 January 2024) was used for visualizing the phylogenetic tree.

### 2.4. Protein Domain, Conserved Motifs, and Gene Structure Analyses

The protein sequences of eleven *GpOSC* genes underwent alignment via the MUSCLE alignment package in MEGA version 7.0. Gene domains were investigated using Batch CD-Search, available at NCBI (https://www.ncbi.nlm.nih.gov/Structure/bwrpsb/bwrpsb.cgi, accessed on 12 January, 2024), with detected domains visualized through TBtools version 2.096 [34]. Conserved motifs within candidate genes were predicted using the MEME program (https://meme-suite.org/meme/tools/meme, accessed on 13 January 2024 [35]), allowing for a maximum of 10 motifs to be identified. Furthermore, intron/exon structures of *OSC* genes were analyzed using TBtools version 2.096. The tertiary structures of GpOSC proteins were predicted using Alphafold (https://alphafold.ebi.ac.uk/, accessed on 13 January 2024), followed by domain analyses conducted across all obtained protein sequences.

### 2.5. Cis-Element Analysis

Promoter sequences, approximately 2000 bp upstream of the start codon for each candidate *GpOSC* gene, were screened for cis-elements. These elements were predicted using PlantCARE (http://bioinformatics.psb.ugent.be/webtools/plantcare/html/, accessed on 15 January 2024) within these regions.

### 2.6. Transcriptome Sequencing and Analyses

In order to investigate the genetic differences among populations of *G. pentaphyllum* distributed in different geographical regions, we collected their wild populations from different areas and cultivated them in a common garden resource nursery under the same temperature, humidity, and soil conditions for over one year. Twelve fresh young leaves from four distinct populations were collected and immediately stored in liquid nitrogen to analyze the expression levels of *GpOSC* genes (Table S2). Total RNA was extracted using an ethanol precipitation protocol combined with a CTAB-PBIOZOL reagent, and RNA quality was quantified with NanoDrop 2000 and Agilent 2100 Bioanalyzer instruments. Stranded RNA libraries were constructed for each sample and sequenced on the Illumina NovaSeq6000 platform to generate paired-end reads of 150 bp (PE150). The raw reads were uploaded into NCBI with SRA accession number PRJNA1186939. SOAPnuke [36] software was employed to filter raw reads, resulting in clean reads that were subsequently mapped onto the reference genome of *G. pentaphyllum* using HISAT2 [37]. Gene expression levels were quantified as fragments per kilobase of transcript per million mapped fragments (FPKM) [38], calculated using the ballgown R package. A heatmap based on log_10_(FPKM+1) data was generated using TBtools version 2.096.

### 2.7. Quantitative Real-Time PCR

According to the results of the transcriptome sequencing analysis, the expression patterns of four differentially expressed *GpOSC* genes (*GpOSC1*, *GpOSC4*, *GpOSC5*, and *GpOSC6*) were further validated using a quantitative reverse transcription polymerase chain reaction (qRT-PCR). Total RNA was subjected to reverse transcription utilizing the PrimeScript RT Reagent Kit (Takara). Primers were designed with Primer3web software version 4.1.0 (https://primer3.ut.ee/, accessed on 8 November 2024), and the *Actin* gene served as a reference (Table S5). The TB Green Premix Ex TaqTM II FAST qPCR kit (Takara) was employed in conjunction with a 25 µL reaction system for the qRT-PCR analysis conducted on a LightCycler 96 instrument (Roche, Beijing, China). Three technical replicates were performed. Finally, the 2^−∆∆Ct^ method was applied to calculate the relative expression levels of *GpOSC* genes.

## 3. Results and Discussion

### 3.1. Genome-Wide Identification and Chromosomal Distribution of GpOSC Family

With the rapid advancement of genome sequencing technology, bioinformatics analysis methods have facilitated the identification of an increasing number of gene families. Previous studies have demonstrated that OSC enzymes play a crucial role in the biosynthesis pathway of triterpenoid saponins, with numerous members of the *OSC* gene family identified and isolated in various plant species. For instance, 13 *OSC* genes have been discovered in *A. thaliana*, 9 of which can be heterologously expressed in yeast [39]. Additionally, 12 *OSC* genes were identified and modified in rice to enhance its triterpenoid content [40]. As more members of the *OSC* gene family are identified, researchers have shifted their focus toward understanding the structure, regulation, and evolution of *OSC* genes [41,42].

Current research has largely elucidated the biosynthetic pathways for ginsenosides. The enzyme-catalyzed conversion of 2,3-oxidized squalene by *DSII* and *PNY* (*β-AS*) genes leads to the production of dammarenediol and β-amyrin, ultimately resulting in the formation of dammarane-type and oleanane-type ginsenosides [43]. Proteins encoded by *OSC* genes represent key initial enzymes involved in gypenoside biosynthesis. Therefore, identifying *OSC* synthesis genes implicated in this pathway is essential for a comprehensive pathway analysis. To identify *GpOSC*s, we conducted BLAST and HMM searches that revealed a total of 11 *GpOSC* genes, which were sequentially designated as *GpOSC1* to *GpOSC11*. Based on annotation data, it was found that these *OSC* genes were located on six chromosomes (Figure S1). Five *GpOSC*s (*GpOSC1*, *GpOSC5*, *GpOSC9*, *GpOSC10*, and *GpOSC11*) were located within telomeric regions on their respective chromosomes; conversely, six other *GpOSCs* (*GpOSC2*, *GpOSC3*, *GpOSC4*, *GpOSC6*, *GpOSC7*, and *GpOSC8*) were clustered in the chromosome arm regions. These results are consistent with previous studies on the *OSC* gene families of *Rosa* and *M. charantia* [17,18]. Chromosomes 2 and 7 each contained three *GpOSC* genes, while chromosomes 4, 8, 9, and 10 each contained one or two *GpOSC* genes.

### 3.2. Physicochemical Property and Subcellular Localization Analysis of GpOSC Family

The basic physicochemical parameters of the GpOSC proteins were calculated. The lengths of the GpOSC proteins ranged from 356 amino acids (GpOSC6) to 767 amino acids (GpOSC11), with molecular weights varying between 40,113.93 Da (GpOSC6) and 88,595.63 Da (GpOSC11). The pIs of these proteins span from 5.90 to 8.08, while the instability index ranges from 40.11 to 50.30 (Table 1). The subcellular localization analysis predicted that the proteins encoded by one gene reside within the peroxisome, four genes were located in the cytoplasm, two genes were associated with the plasma membrane, and four genes were found in lysosomes. These findings are similar to previously reported *OSC* genes in *Astragalus membranaceus* [44], but differ from those observed in *Chenopodium quinoa* [45]. In *C. quinoa*, among the fifteen *CqOSC* genes identified, all except for *CqOSC2*, which is located both in the cytoplasm and chloroplast, are exclusively situated within the chloroplast [45].

### 3.3. Phylogenetic Analysis and Classification of OSC Gene Family

**Figure 1:**
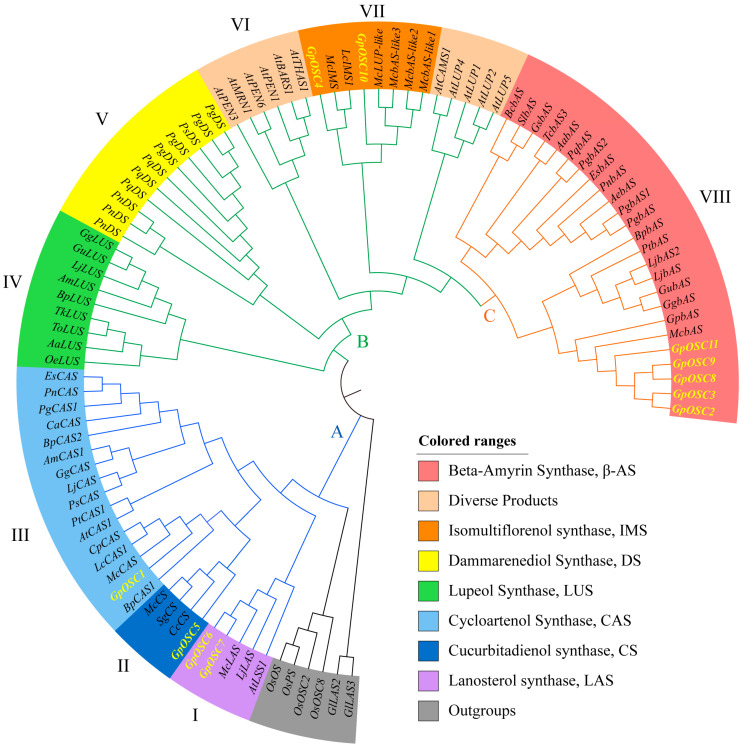
**Phylogenetic analysis of 95 *OSC* genes of different plant species.** Groups are distinguished with different colors and *OSC*s of *G. pentaphyllum* (*GpOSC*s) are highlighted. Information on plant species and corresponding *OSC*s is listed in Table S1.

To further elucidate the evolutionary relationship of the *OSC* gene family in *G. pentaphyllum*, a phylogenetic analysis was conducted on the *GpOSC* genes and those of other reported dicotyledonous plants, including *A. thaliana*, *P. ginseng*, and *M. charantia* (Table S1). The *OSC* genes from the monocotyledonous plant *Oryza sativa* and the fungus *Ganoderma lucidum* were used as outgroups. The results indicated that all *OSC* genes could be divided into eight distinct subfamilies (Figure 1). Upon a further analysis of various branches within the phylogenetic tree, it was observed that synthase genes encoding cycloartenol, lanosterol, and cucurbitadienol formed Group A, which included *GpOSC6*, *GpOSC7*, *GpOSC1*, and *GpOSC5*. Group B comprised gene branches responsible for encoding lupeol synthase, dammarenediol synthase, and other functions, with *GpOSC10* and the *β-AS-like* genes from *M. charantia* clustering together, while *GpOSC4* was identified as an *IMS* gene. Genes encoding β-amyrin synthase (β-AS) were classified into Group C; within this group, *GpOSC11*, *GpOSC9*, *GpOSC8*, *GpOSC3*, and *GpOSC2* formed monophyletic branches.

Previous studies have suggested that cycloartenol synthase (*CAS*) and lanosterol synthase (*LAS*), both involved in sterol synthesis processes, may represent ancestral genes that evolved either directly or indirectly from *OSC* genes [40,46]. For instance, lower plants such as *Chlamydomonas reinhardtii* and mosses contain only a single type of *OSC* for sterol synthesis, whereas higher plants exhibit a broader diversity of *OSC* types (approximately 9–16). Moreover, additional phylogenetic studies have indicated that the expansion of OSC genes in higher plants primarily arose from their ancestor *LAS* through processes such as tandem duplication events, positive selection pressures, and divergent evolution [47], which is consistent with our findings.

Interestingly, our phylogenetic results revealed that the *GpOSC* genes did not cluster within the same clade as the *DS* genes of species belonging to the genus *Panax.* This finding is consistent with a recent study identifying an *OSC* gene that is co-expressed with genes involved in the triterpenoid synthesis pathway of *G. pentaphyllum* [48]. Through the heterologous expression of a *GpOSC* gene in brewing yeast and subsequent functional validation, it was discovered that this gene could encode dammarenediol synthase, which aligns with the DSII function identified in ginseng, catalyzing the conversion of 2,3-oxidized squalene to dammarenediol. Therefore, we propose that the genes encoding dammarenediol synthase in *G. pentaphyllum* have undergone independent convergent evolution with *DS* genes, endowing them with dammarenediol synthase activity and leading to the production of similar types of saponins across two distinct plant families. Additionally, the biosynthesis of dammarane-type saponins in *G. pentaphyllum* may involve more complex synthetic pathways that warrant further investigation.

In conclusion, a total of eleven *GpOSC* genes were identified within the genome of *G. pentaphyllum*. However, these candidate genes did not include any *DS* or *LS* genes. Therefore, we speculate that *GpOSC* genes may possess multiple functions akin to certain *OSC* genes from other species. For instance, *TwOSC1* from *Tripterygium wilfordii* can cyclize 2,3-oxidized squalene to produce β-amyrin, α-amyrin, and friedelin [49], while *BfOSC1* from *Bauhinia forficata* can similarly cyclize 2,3-oxidized squalene to yield β-amyrin, α-amyrin, and lupeol [50].

### 3.4. Protein Domain, Conserved Motifs, and Gene Structure of GpOSC Proteins

**Figure 2:**
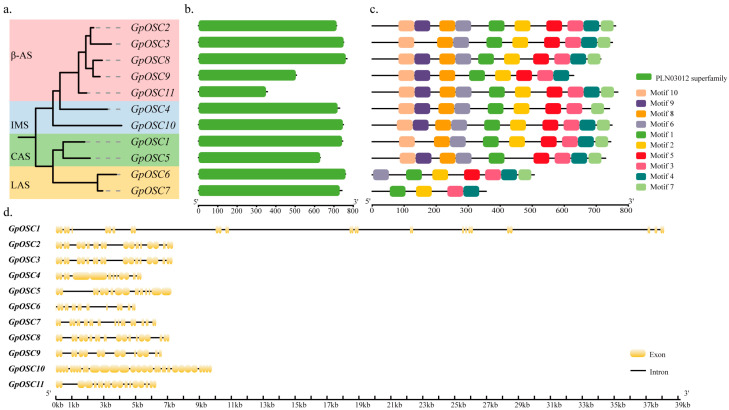
Phylogenetic relationships (**a**), protein domain (**b**), conserved motifs (**c**), and gene structure (**d**) among 11 GpOSC proteins.

The proteins of the OSC family exhibit highly conserved domains and significant amino acid homology. Studies have shown that the homology among CAS enzymes can reach as high as 80% [23]. To analyze the protein domains of GpOSC proteins, a phylogenetic tree was constructed based on their protein sequences (Figure 2a). This analysis revealed a highly conserved protein domain within the GpOSC proteins, specifically identifying only one conserved domain: the PLN03012 superfamily (Figure 2b).

Additionally, both conserved and unique motifs in GpOSC proteins were also identified (Figure 2c). From an overarching perspective, it was observed that motifs tended to be similar among genes located on the same branch or exhibiting close evolutionary relationships (Figure S2). Most GpOSC proteins contained between 8 and 10 motifs; however, GpOSC6 and GpOSC7 exhibited fewer motifs, with GpOSC7 containing only four motifs. It is speculated that the loss of conserved motifs may impact protein functionality. The conserved sequences DCTAE and QW (QXXXXXW) were present in motif 2, motif 9, and motif 10; notably, all eleven GpOSC protein sequences contained these specific motifs (Figure S3). A multiple sequence alignment of the eleven GpOSC proteins revealed numerous homologous sequences within these proteins (Figure S4). Previous studies have demonstrated that OSC family proteins have relatively high amino acid similarity, and their amino acid sequences generally contain a DCTAE motif along with several QW conserved motifs [17]. The D and C residues within the DCTAE motif were confirmed to play a role in initiating the OSC cyclization reaction. Meanwhile, the QW motif is crucial for stabilizing carbon cation intermediates while maintaining structural integrity during cyclization [51,52]. Additionally, pentacyclic triterpenoid OSC proteins also contain a conserved sequence designated as MWCYCR; herein lies an important function for both W and Y residues in stabilizing the D/E ring structure. The comprehensive results indicated that although the protein sequences of most members of the *GpOSC* gene family have undergone changes throughout the evolutionary process, the motifs remain highly conserved. These findings underscore the importance of motifs in determining the specificity of GpOSC proteins.

The bioinformatics analysis of the *T. wilfordii OSC* gene family revealed that alterations in conserved motifs at either the N-terminus or C-terminus could significantly impact the physiological function of these proteins [53]. More than 100 types of OSC products can be synthesized in plants, and previous studies have demonstrated that modifications to the amino acid sequence of OSC proteins can lead to variations in synthesized products. The diversity found within core sites among OSC enzymes contributes to structural variability within the *OSC* gene family. A previous study identified that Trp259 in a β-AS enzyme regulates β-amyrin production, and mutation from this residue to Tyr261 can result in a conversion between tetracyclic and pentacyclic triterpenes [15].

The MEME and TBtools software were employed to illustrate the gene structures across various *GpOSC* members. The analysis revealed significant differences in gene structure among members of the *GpOSC* family (Figure 2d). In terms of sequence length, *GpOSC1* exhibited the longest sequence, while *GpOSC6* had the shortest. Additionally, notable discrepancies were observed regarding exon and intron counts; specifically, exon numbers ranged from 18 to 36 and intron counts varied from 8 to 17. Most members that clustered on similar branches or within close evolutionary distances displayed comparable structures as well as similar sequence lengths and exon/intron numbers (e.g., *GpOSC2* and *GpOSC3*). However, substantial differences were also observed among members situated on identical branches. For instance, while both *GpOSC7* and *GpOSC9* shared similar sequence lengths, their respective exon and intron counts differed significantly. Conversely, while there are notable differences between *GpOSC1* and *GpOSC5* regarding sequence length, their exon/intron structure is comparable along with a highly similar coding sequence (CDS) region. Based on this similarity concerning evolutionary distance as well as CDS characteristics, it can be inferred that these genes might fulfill analogous biological functions.

In recent years, there has been substantial growth in utilizing saponin active ingredients within pharmaceuticals; market demand continues to rise annually. Consequently, enhancing saponin content within medicinal plants has emerged as a pivotal area for research endeavors. Investigations have shown that exon deletions present within *DS* genes, key enzymes responsible for catalyzing dammarane-type saponins found within the *Aralia elata* genome, lead to an incapacity for synthesizing such compounds [54]. Thus, comprehending how members from the *OSC* gene family precisely regulate both initiation/termination processes during saponin synthesis reactions alongside ensuring stability among intermediate products, and managing product ratios throughout catalytic mechanisms, remains an imperative field requiring further exploration.

### 3.5. Cis-Element Prediction of GpOSCs

Cis-elements, including promoters, enhancers, regulatory sequences, and inducible elements, play crucial roles, governing gene expression while also being integral components influencing post-transcriptional modifications [55]. To investigate how upstream sequences associated with *GpOSC* genes affect their expression levels, cis-element prediction was carried out on the 2000 bp upstream region of 11 *GpOSC* gene promoters (Figure S5). The analysis revealed significant differences in the upstream elements of the *GpOSC* gene family, showcasing a wide diversity of regulatory elements. These include light-responsive elements, hormone-responsive elements (such as auxin, abscisic acid, gibberellin, methyl jasmonate, etc.), various abiotic stress-responsive elements (including drought, low temperature, and anaerobic conditions), and transcription factor-binding sites, such as MYB binding sites. Researchers have found that cis-elements are closely associated with gene function, and these elements align with the biological functions of GpOSC genes in response to stress and organismal injury [56]. Therefore, it was hypothesized that *G. pentaphyllum* might regulate *GpOSC* gene expression synergistically through various hormone response elements, such as auxin (TGA), salicylic acid (TCA), abscisic acid (ABA), gibberellin (GA), and methyl jasmonate (MeJA). This regulation could also involve stress response elements related to anaerobic conditions, drought, and low temperatures. Such interactions may influence saponin synthesis and accumulation in response to environmental stresses. Additionally, MYB binding sites were identified within the promoter region of *GpOSC*, similar to observations made in *C. quinoa* [45], suggesting that MYB transcription factors might play a role in regulating the expression of *GpOSC* genes. Numerous hormone-responsive elements were also detected in the upstream regions of *CqOSC* in *C. quinoa*, particularly MeJA-responsive elements, which constituted a substantial proportion. In *C. quinoa*, soaking leaves in MeJA for 30 s was found to enhance saponin synthesis [57], potentially offering valuable insights for future experimental endeavors.

### 3.6. Expression Level of GpOSCs Among Different Populations

Numerous studies have demonstrated that the highest content of gypenosides in *G. pentaphyllum* is predominantly found in the leaves [9]. Additionally, key enzyme genes involved in the gypenoside biosynthesis pathway exhibit relatively high expression levels in both stems and leaves [30]. Therefore, in our study, we collected *G. pentaphyllum* plants from various regions and cultivated them in a common garden setting. Twelve young leaves from four distinct populations were collected to analyze the expression levels of *GpOSC* genes based on RNA sequencing (Tables S3 and S4), and experimentally validated using qRT-PCR. A heatmap illustrating the expression patterns of 11 *GpOSC* genes across different populations was generated (Figure S6). The results indicated that five β-AS genes (*GpOSC2*, *GpOSC3*, *GpOSC8*, *GpOSC9*, and *GpOSC11*) along with one β-AS-like gene (*GpOSC10*) exhibited consistently low expression levels across all populations. Notably, *GpOSC1*, a *CAS* gene, showed the highest expression level among populations except for the QC population. The expression levels of *GpOSC4*, *GpOSC5*, and *GpOSC6* showed significant variation among different populations. From a population perspective, *GpOSC1* was highly expressed within the DH population, followed by elevated expressions of both *GpOSC4* and *GpOSC5*. In contrast, high expressions of both *GpOSC1* and *GpOSC6* were observed in XA and ZJ populations; however, the expression levels of all *GpOSC* genes were relatively low in the QC population (Figure S6). Correspondingly, the results of qRT-PCR showed a consistent result that the DH population had the highest expression level across *GpOSC1*, *GpOSC4*, and *GpOSC5*, while the *GpOSC6* gene was highly expressed in XA and ZJ populations (Figure S7).

A study on *Citrus grandis* integrated transcriptome and metabolome data to identify key medicinal components that significantly varied across different growing regions while conducting association analyses with differentially expressed functional genes. This approach provided valuable insights into quality differences observed in *C. grandis* sourced from diverse regions [58]. Similarly, analyses of RNA-seq data from *Citrus* reticulata cultivated in various regions revealed that transcripts associated with monoterpene biosynthesis were more prevalent in the core areas. Furthermore, it was also demonstrated that genes responsive to biotic and abiotic stress exhibited a correlation with monoterpene production in these regions, suggesting that unique soil properties and microbial environments may affect monoterpene accumulation [59]. Consequently, it was speculated that *G. pentaphyllum* distributed across different regions might exhibit significant variations in the expression of members of the *OSC* gene family due to genetic differences or environmental factors such as soil conditions. This could lead to the synthesis of diverse types of gypenosides in *G. pentaphyllum* from various regions, which underpins the authenticity of *G. pentaphyllum* as a traditional Chinese medicinal herb. Therefore, investigating the expression patterns of *OSC* gene family members in *G. pentaphyllum* from different regions could elucidate the underlying mechanisms responsible for quality discrepancies and provide a foundation for cultivating, producing, and breeding high-quality varieties of *G. pentaphyllum*. This would contribute to the sustainable development of high-quality traditional Chinese medicine. However, further studies on gypenoside content and genetic evidence remain necessary through biochemical molecular genetics and multi-omics approaches, including metabolomic analyses in the future.

## 4. Conclusions

In the present study, we employed bioinformatics analysis methods and qRT-PCR experimental validation to identify 11 *OSC* genes within the genome of *G. pentaphyllum*. We explored their physicochemical properties, conserved domains, gene structures, phylogenetic relationships, and expression levels across four geographically distinct populations. The results indicated that the *GpOSC* genes exhibited conservation in both structure and evolutionary patterns. Notably, findings from the phylogenetic tree suggested that the genes encoding dammarenediol synthase in *G. pentaphyllum* have undergone independent convergent evolution alongside the *DS* genes of the *Panax* genus. This convergence has led to the acquisition of dammarenediol synthase activity and ultimately resulted in the production of similar types of saponins across these two different plant families. Furthermore, this study identified significant variations in *OSC* gene family expression among the four populations examined. These differences suggest that *G. pentaphyllum* may synthesize different types of gypenosides depending on inherent genetic variations or external factors such as environmental conditions and soil composition. In summary, this study enhances our understanding of the systematic evolution of *OSC* genes in *G. pentaphyllum*, as well as elucidating biosynthetic mechanisms underlying triterpenoid compounds. This is particularly significant for exploring molecular mechanisms responsible for forming dammarane-type triterpenoid saponins and addressing geographical differences observed within *G. pentaphyllum*. In future studies, plant genetic engineering technologies could be employed to further investigate correlations between expression differences among *GpOSC* family members and various saponin types, providing a theoretical foundation to support advancements in saponin metabolism engineering, standardized cultivation practices, variety improvement efforts, and sustainable resource development related to *G. pentaphyllum*.

## Figures and Tables

**Table 1:** Information of 11 *OSC* gene family members in *G. pentaphyllum*.

Gene Name	Sequence ID	Number of Amino Acids	Molecular Weight	Theoretical pI	Instability Index	Aliphatic Index	Grand Average of Hydropathicity	Subcellular Localization Prediction
***GpOSC1***	Gype02G0020.1	745.00	84,682.54	5.90	40.50	84.62	−0.27	Peroxisomal
***GpOSC2***	Gype02G0431.1	760.00	87,741.97	6.75	47.81	78.68	−0.31	Cytoplasmic
***GpOSC3***	Gype02G0505.1	750.00	86,268.80	8.08	45.64	81.81	−0.26	Cytoplasmic
***GpOSC4***	Gype04G0170.1	741.00	85,668.05	6.23	49.61	76.36	−0.35	Plasma Membrane
***GpOSC5***	Gype04G1624.1	729.00	83,516.29	6.48	47.25	83.61	−0.33	Lysosomal
***GpOSC6***	Gype07G1533.1	356.00	40,133.93	6.46	42.66	87.33	−0.24	Lysosomal
***GpOSC7***	Gype07G1535.1	506.00	57,889.42	7.55	40.11	83.24	−0.26	Lysosomal
***GpOSC8***	Gype07G2694.1	714.00	82,651.78	6.40	43.24	75.28	−0.35	Lysosomal
***GpOSC9***	Gype08G0091.1	629.00	72,210.32	5.91	42.17	81.29	−0.32	Cytoplasmic
***GpOSC10***	Gype09G0155.1	749.00	86,355.59	6.61	47.20	77.86	−0.37	Plasma Membrane
***GpOSC11***	Gype10G1930.1	767.00	88,595.63	6.36	50.30	75.70	−0.37	Cytoplasmic

## Data Availability

The raw reads of RNA sequencing were uploaded into NCBI with SRA accession number PRJNA1186939. Data supporting the reported results can be obtained from the corresponding author.

## Supplementary Materials

The following supporting information can be downloaded at: https://www.mdpi.com/article/10.3390/life14121599/s1, Figure S1: Chromosomal localization of *OSC* gene family members of *G. pentaphyllum*; Figure S2: Tertiary structure predictions of the 11 candidate GpOSC proteins; Figure S3: Multiple sequence alignment of the eleven GpOSC proteins; Figure S4: Sequence logos of the top ten significantly enriched motifs; Figure S5: Visualization of promoter cis-element prediction of *OSC* genes within *G. pentaphyllum*; Figure S6: Heatmap of gene expression values in different populations of *G. pentaphyllum*; Figure S7: The expression levels of four *GpOSC* genes in four distinct populations investigated using qRT-PCR; Table S1: Informations of 84 *OSC* genes used in phlogenetic tree analysis; Table S2: Informations of samples used in transcriptom analysis; Table S3: RNA-seq data used for transcriptome analysis; Table S4: The FPKM values of *GpOSC* genes in 12 samples from different populations of *G. pentaphyllum*; Table S5: Primers of four GpOSC genes in *G. pentaphyllum*.
