# Supplementary material for: Genome-Wide Identification and Characterization of OSC Gene Family in Gynostemma pentaphyllum (Cucurbitaceae)
Source: Life (Basel). 2024 Dec 4;14(12):1599. doi: 10.3390/life14121599 (PMC11676476; doi:10.3390/life14121599)
Supplement: Supplementary file 1 [file life-14-01599-s001.zip › legends-v3.pdf]

Figure Legends

Figure 1. Phylogenetic analysis of 95 *OSC* genes of different plant species. Groups were distinguished with different colors and *OSC*s of *G. pentaphyllum* (*GpOSC*s) were highlighted in yellow. The information on plant species and corresponding *OSC*s are listed in Table S1.

Figure 2. Phylogenetic relationships (a), protein domain (b), conserved motifs (c) and gene structure (d) among 11 GpOSC proteins.

Figure S1. Chromosomal localization of *OSC* gene family members of *G. pentaphyllum*.

Figure S2. Tertiary structure predictions of the 11 candidate GpOSC proteins.

Figure S3. Multiple sequence alignment of the eleven GpOSC proteins. The red and orange boxes in the figure are the conserved sequences DCTAE and QXXXXXW of the *OSC* gene family.

Figure S4. Sequence logos of the top 10 significantly enriched motifs.

Figure S5. Visualization of promoter cis-element prediction of *OSC* genes in *G. pentaphyllum*. Blocks of different colors represent different elements.

Figure S6. Heatmap of gene expression values in different populations of *G. pentaphyllum* generated based on the normalized $\log_{10}$(FPKM+1) represented by the blue red gradation.

Figure S7. The expression levels of four *GpOSC* genes in four distinct populations investigated using qRT-PCR.
