# Supplementary figures and images for: Genome-Wide Identification and Characterization of OSC Gene Family in Gynostemma pentaphyllum (Cucurbitaceae)
Source: Life (Basel). 2024 Dec 4;14(12):1599. doi: 10.3390/life14121599 (PMC11676476; doi:10.3390/life14121599)

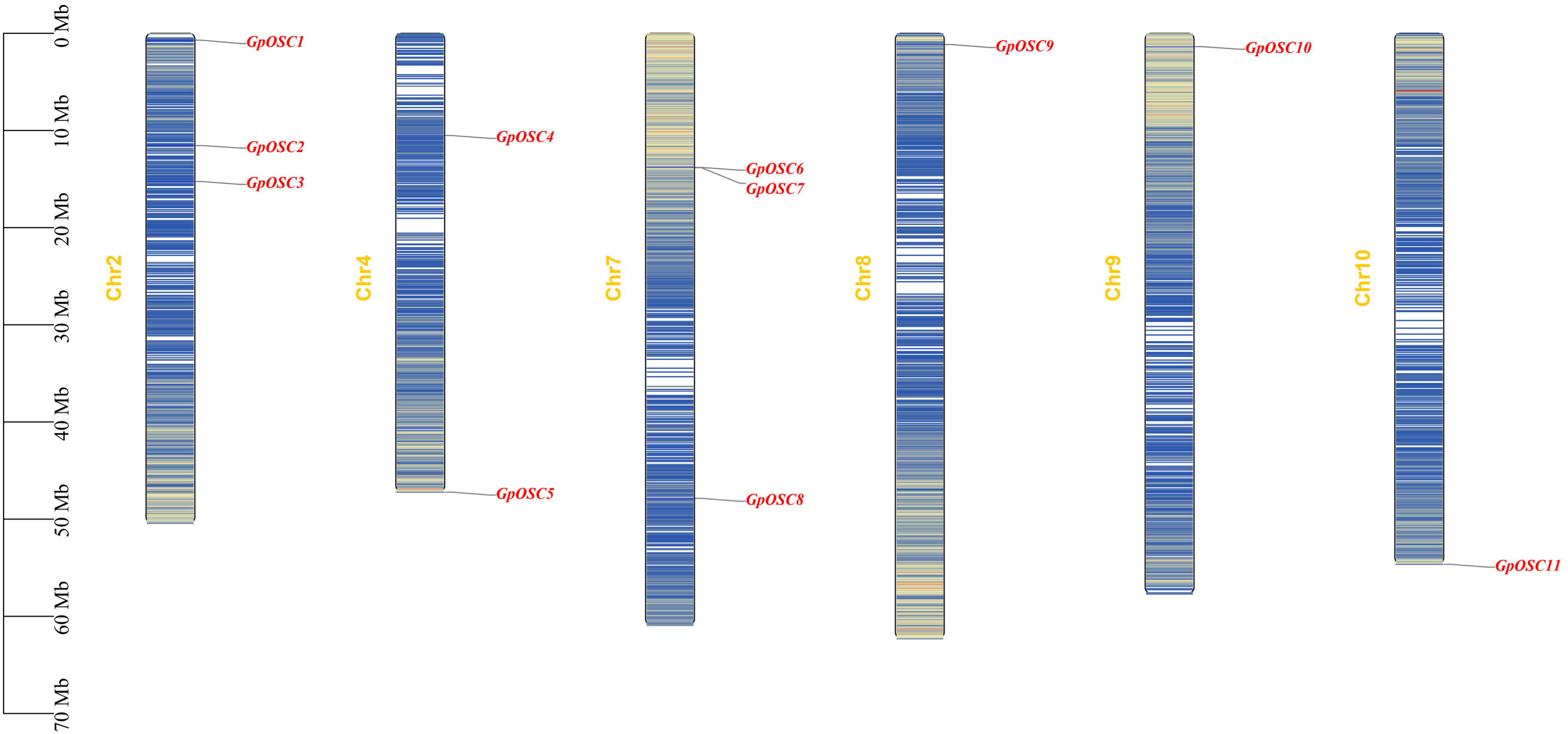

Supplement: Supplementary file 1 [file life-14-01599-s001.zip › Figure S1.pdf]

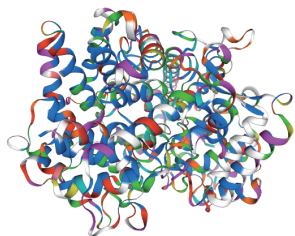

*GpOSC1*

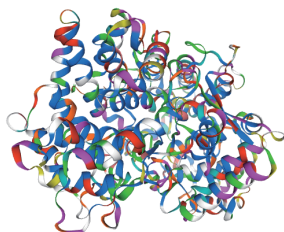

*GpOSC2*

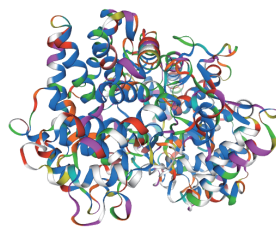

*GpOSC3*

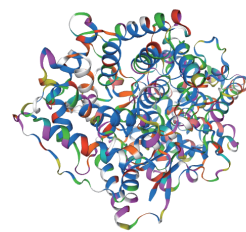

*GpOSC4*

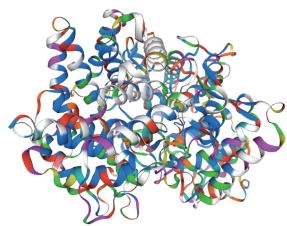

*GpOSC5*

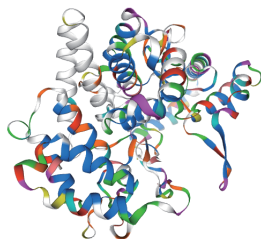

*GpOSC6*

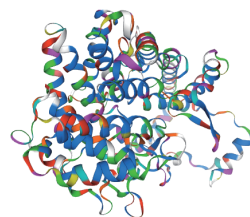

*GpOSC7*

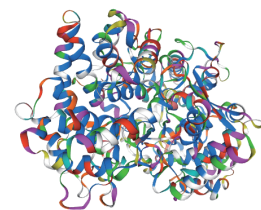

*GpOSC8*

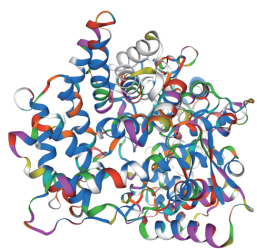

*GpOSC9*

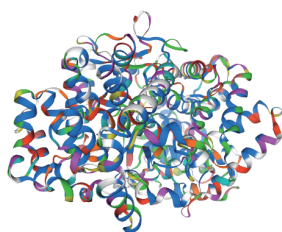

*GpOSC10*

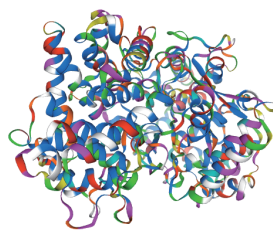

*GpOSC11*

Supplement: Supplementary file 1 [file life-14-01599-s001.zip › Figure S2.pdf]

1.

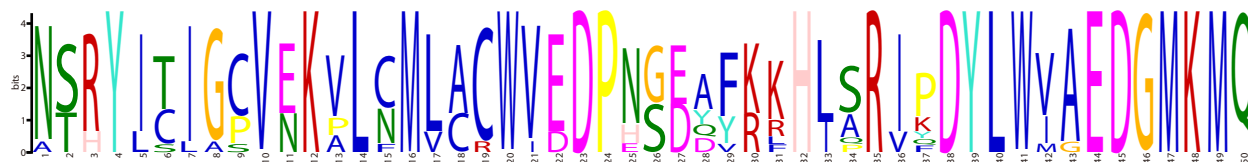

2.

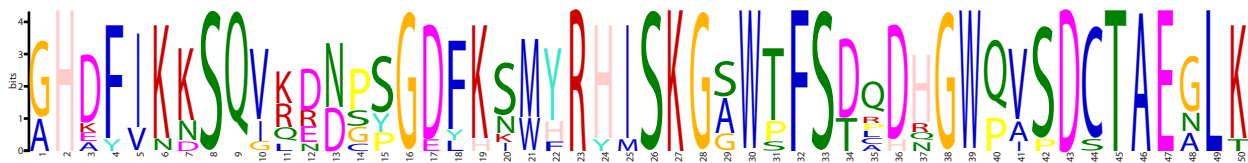

3.

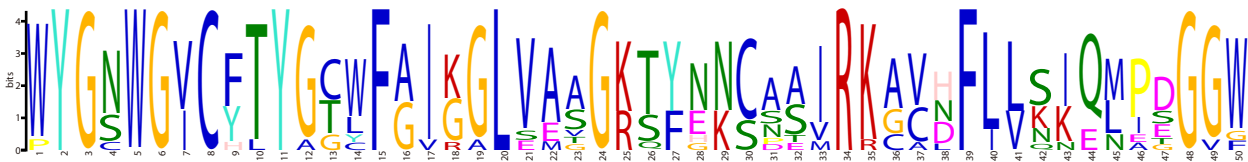

4.

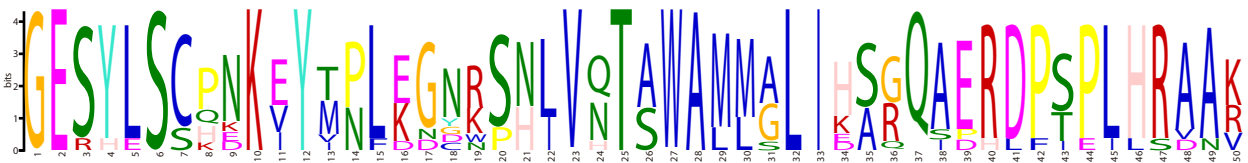

5.

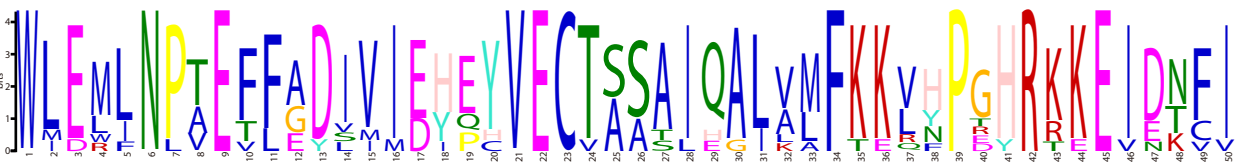

6.

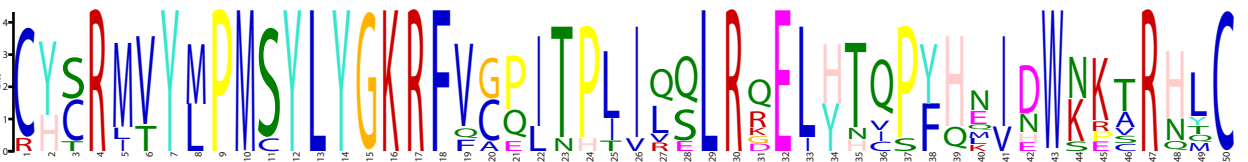

7.

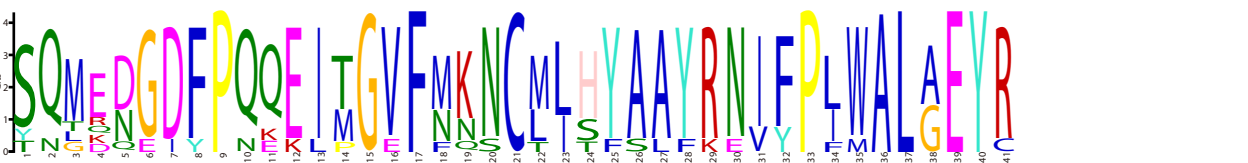

8.

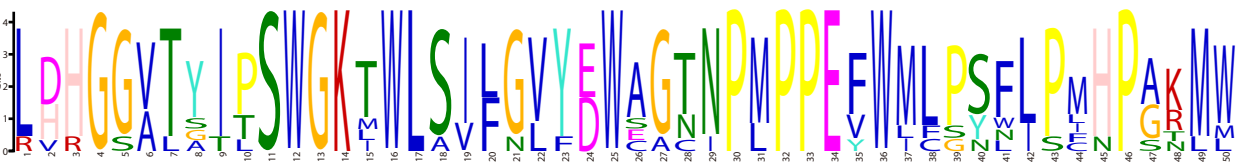

9.

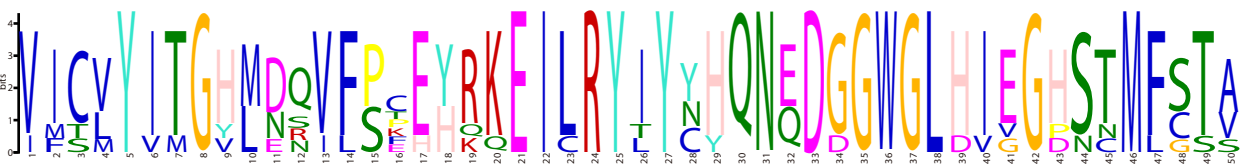

10.

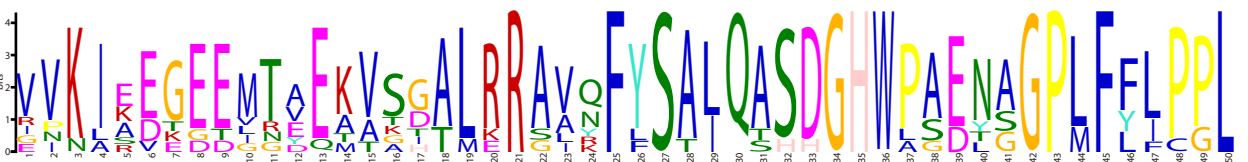

Supplement: Supplementary file 1 [file life-14-01599-s001.zip › Figure S3.pdf]

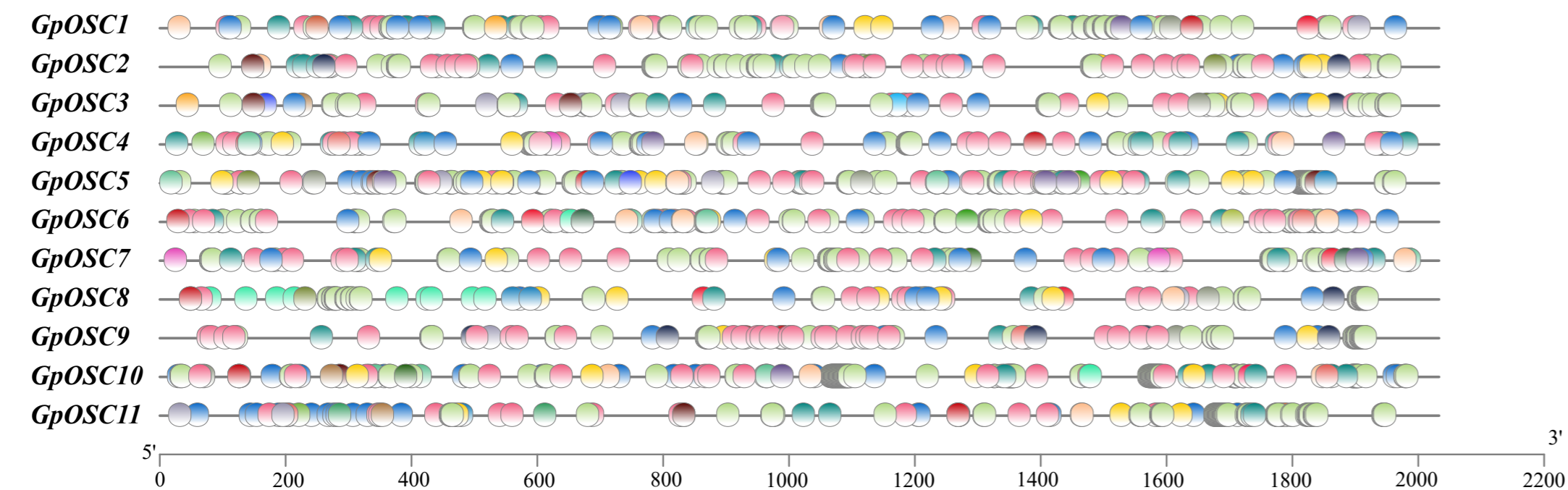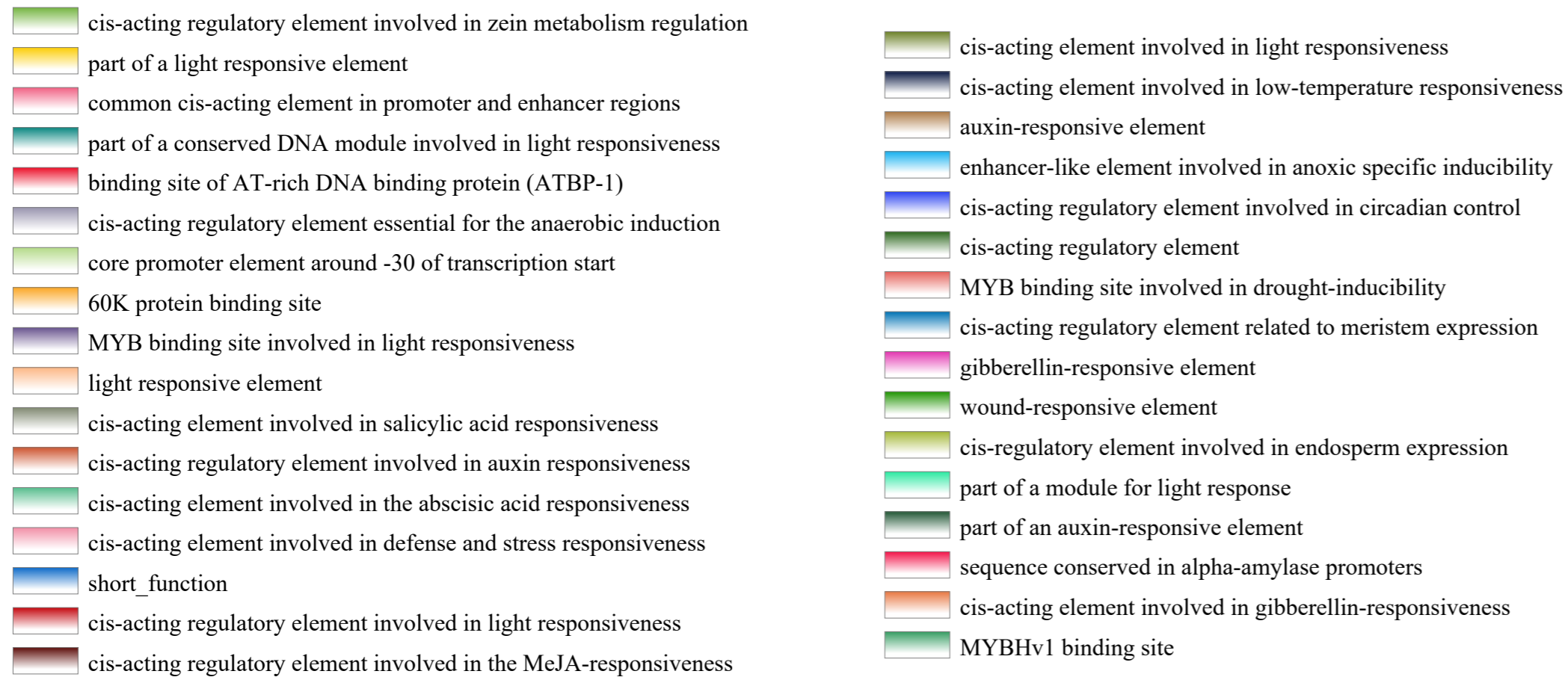

Supplement: Supplementary file 1 [file life-14-01599-s001.zip › Figure S5.pdf]

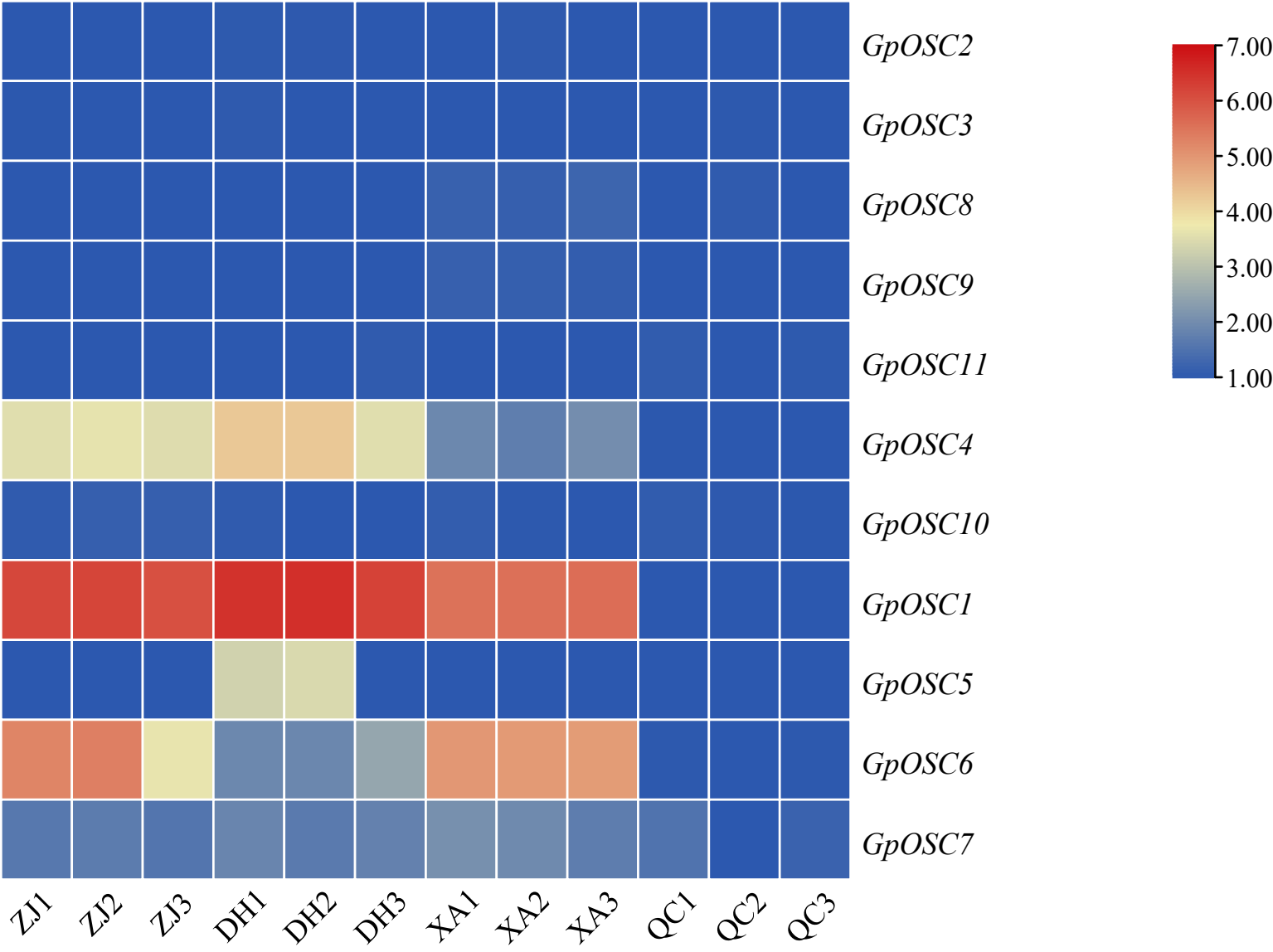

Supplement: Supplementary file 1 [file life-14-01599-s001.zip › Figure S6.pdf]

a.

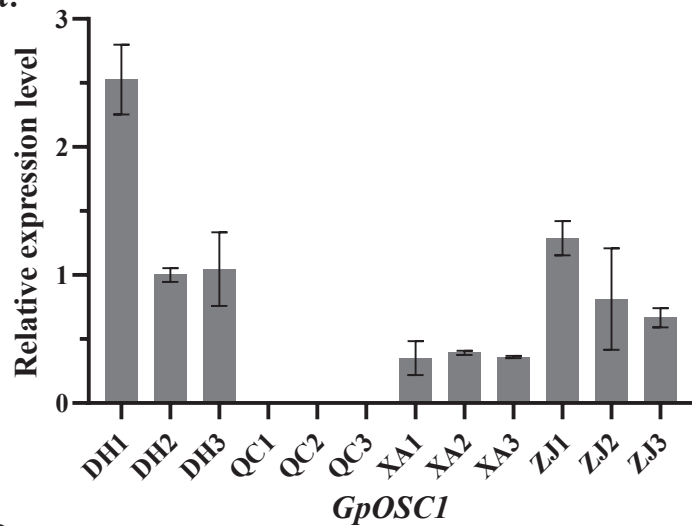

b.

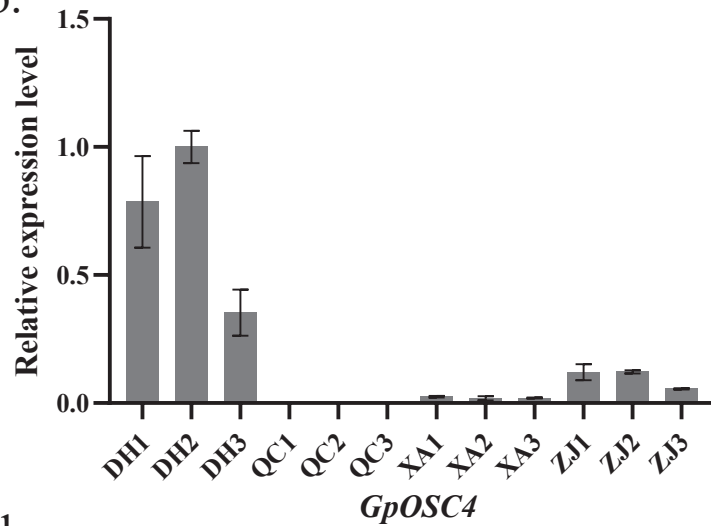

c.

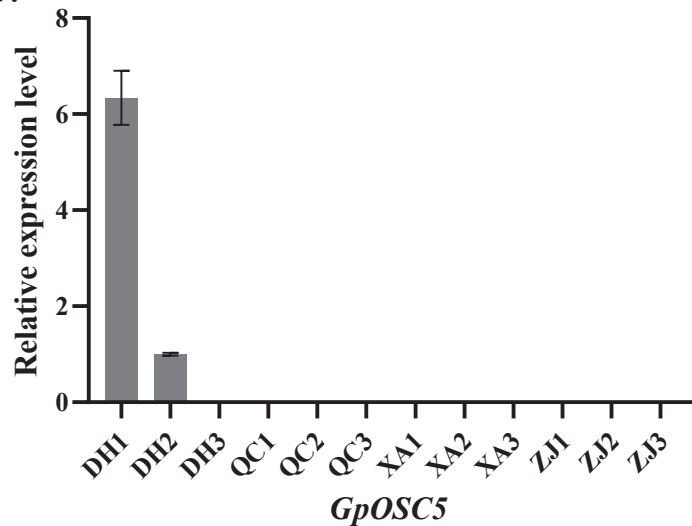

d.

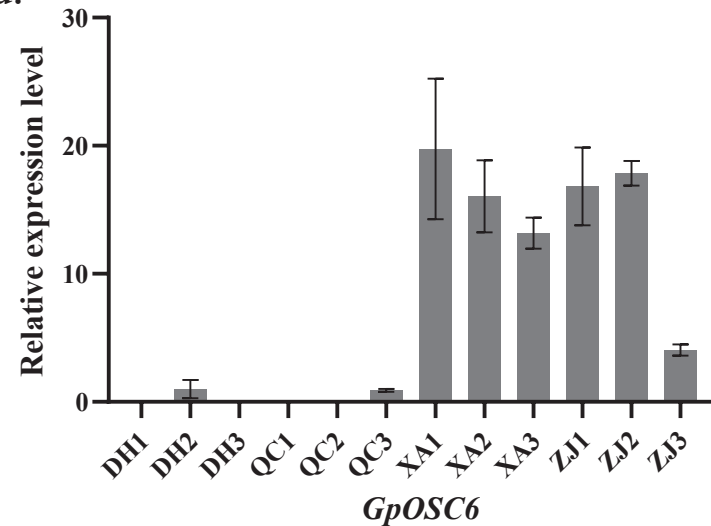

Supplement: Supplementary file 1 [file life-14-01599-s001.zip › Figure S7.pdf]
